# Artificial intelligence in cardiovascular practice

**DOI:** 10.1097/01.JAA.0000000000000204

**Published:** 2025-04-24

**Authors:** Marci Farquhar-Snow, Amy E. Simone, Sheel V. Singh, Reamer L. Bushardt

**Affiliations:** **Marci Farquhar-Snow** is a retired assistant professor, formerly practicing in the Department of Cardiovascular Medicine at Mayo Clinic College of Medicine and Science in Scottsdale, Ariz. **Amy E. Simone** is a consultant at Edwards Lifesciences in Burlingame, Calif. **Sheel V. Singh** is a second-year student in the PhD program in Health and Rehabilitation Sciences at Massachusetts General Hospital Institute of Health Professions in Boston, Mass. **Reamer L. Bushardt** is provost and vice president for academic affairs and a professor at Massachusetts General Hospital Institute of Health Professions, as well as a research associate in the Department of Physical Medicine and Rehabilitation at Harvard Medical School in Boston, Mass. Marci Farquhar-Snow serves on the Cardiovascular Team Editorial Board at the *Journal of the American College of Cardiology*. Amy E. Simone is chair-elect, CV Team Section Leadership Council, American College of Cardiology, and founder of JC Medical. Reamer L. Bushardt is editor-in-chief emeritus of *JAAPA*. The authors have disclosed no other potential conflicts of interest, financial or otherwise.

**Keywords:** advanced practice providers, AI, artificial intelligence, cardiovascular disease, machine learning, robotics

## Abstract

Artificial intelligence (AI) is everywhere, but how is this expansive technology being used in cardiovascular care? This article explores common AI models, how they are transforming healthcare delivery, and important roles for clinicians, including advanced practice providers, in the development, adoption, evaluation, and ethical use of AI in cardiovascular care.

**Figure:**
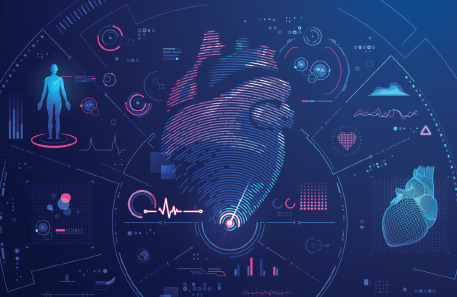
No caption available.

**Box 1:**
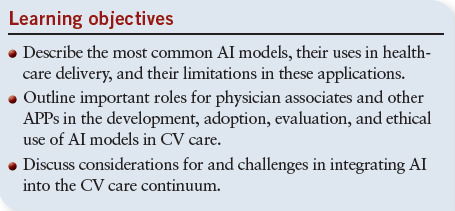
No caption available.

Artificial intelligence (AI) is everywhere these days, but how is this expansive technology being used in the healthcare of individuals with cardiovascular (CV) disease? What do advanced practice providers (APPs), including physician associates and advanced practice registered nurses, need to know about AI to ensure it advances safe, effective, and equitable care?

AI is a type of technology associated with computers and other machines that enables these devices to imitate or mimic human capabilities.^1^ Through adequate training, machines can identify recurring patterns and autonomously learn rules that facilitate their use in various clinical situations and tasks throughout the care continuum, from screening to diagnosis to management. Will AI replace healthcare professionals, such as APPs or physicians? Though this is a provocative question, AI is not fundamentally designed or developed to replace clinicians. It is designed, however, to repurpose roles, support complex analyses, and improve efficiency.^2^

This article describes and explores common AI models and how they are transforming healthcare delivery. It also outlines important roles for APPs in the development, adoption, evaluation, and ethical use of these AI models in CV care, pointing them to recent reports that highlight the utility and application of AI technologies in the care of individuals and populations with CV disease.^3^-^6^ Following its exploration of AI models, this article presents a novel schema for incorporating AI-infused technologies into the care continuum that considers patient perspectives, shared decision-making, and interprofessional collaborative practice. Two cases from clinical practice highlight how AI is currently being used in CV care, with considerations for how APPs are or can be involved in the medical, procedural, and surgical aspects of care. As AI-driven systems become more integrated into various workflows across healthcare organizations, it is crucial for APPs to understand these systems' capabilities, limitations, and challenges. Finally, we offer a call to action for APPs to be leaders and change agents in the ethical and responsible adoption of AI in clinical practice, where it can promote safe, effective, equitable, and high-quality care.

**Box 2:**
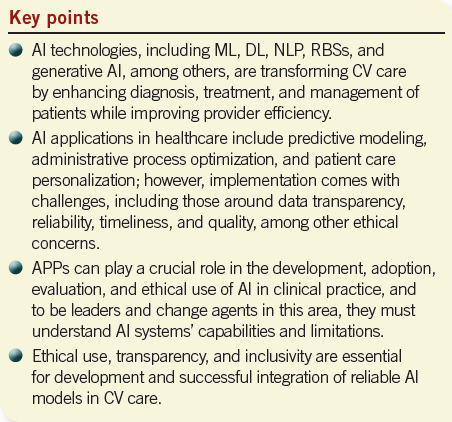
No caption available.

**Box 1** contains *Case study #1*, which provides a starting point for this article's discussion of AI models, how they can be incorporated into clinical practice, and ways in which they can promote enhanced patient care.

## AI MODELS

### Machine learning (ML)

ML is a subfield of AI that enables computers to analyze large sets of data autonomously, uncover patterns within datasets, apply patterns to new data, and execute computational tasks with greater efficiency than humans.^7^ ML can be used to reveal underlying disease mechanisms and enhance the accuracy of diagnosis, treatment, and risk prediction through its identification of clinically significant patterns or phenotypes that may not be readily discernible.^8^ For example, the management of heart failure (HF) often involves complex diagnostic and treatment options that require clinicians to sort through extensive, voluminous clinical information. Using traditional analytical methods, an APP encountering a patient with a new shortness of breath symptom may struggle to apply multiple algorithms and evidence-based research findings regarding specific CV diagnoses and non-CV comorbidities, such as hematologic or maternal health conditions, for precision patient management. Alternatively, by using ML to analyze large datasets, the APP could increase efficiency and accuracy in HF diagnosis and optimize the condition's management.^9^

ML algorithms can assist APPs in selecting an optimal drug therapy regimen. Jing and colleagues developed a population management strategy for patients with HF (N = 26,971) by harnessing large clinical datasets.^10^ Using Geisinger electronic health record (EHR) data, ML models were trained to predict 1-year all-cause mortality. The model identified actionable care gaps, meaning that the gaps could potentially be addressed or improved: these included influenza vaccination, BP of less than 130/80 mm Hg, A1C of less than 8%, cardiac resynchronization therapy, and active medications (angiotensin-converting enzyme inhibitor/angiotensin II receptor blocker/angiotensin receptor-neprilysin inhibitor, aldosterone receptor antagonist, hydralazine, and evidence-based beta-blocker).^10^ To determine if the identified care gaps were associated with higher mortality, ML methods were used to close these gaps (assuming the gaps were fixed in their model), while keeping all other variables unchanged. The change in risk score was calculated for the baseline study population and compared with the ML-adjusted model to estimate the reduction in estimated mortality and the number of lives that could be saved.^10^ Out of the 13,238 currently living patients in the study, 2,844 were predicted to die within 1 year; however, by applying ML to close the evidence-based care gaps, more than 230 of these lives were predicted to be saved. This study showed that ML has promising predictive performance that may outperform traditional logistic regression models.

There is an art to building reliable ML models. The process involves training algorithms to identify patterns in the data, extract relevant insights, and produce accurate predictions or classifications. This critical training step enables ML models to learn from data and generalize new knowledge that can be used in practice.

### Deep learning (DL)

DL is a subset of ML that employs multiple layers of artificial neural networks to uncover or forecast patterns.^11^ DL is particularly advantageous in managing extensive data within the EHR with multiple data sources, since it can adapt with little to no human intervention. Multiple platforms that interpret echocardiograms, identify ECG heart rhythms, and detect cardiac diseases from surface ECGs can be used in risk prediction. Using these features, DL can generate new variables that are not in the training set to enhance efficiency for data analysis and transformation.^9^ For example, Golas and colleagues demonstrated that DL methods can surpass conventional techniques when predicting 30-day readmission rates among patients with HF.^12^ Kwon and colleagues also demonstrated that a DL algorithm could outperform existing scoring systems, such as the Get with the Guidelines-Heart Failure (GWTG-HF) score and Meta-Analysis Global Group in Chronic (MAGGIC) Heart Failure score, by accurately predicting both in-hospital and long-term mortality.^13^ Notably, clinicians may be hesitant to adopt an AI-recommended diagnosis if the rationale and evidence are unclear.^14^ To illustrate, Han and colleagues showed that imperceptible interference on ECG waveforms, known as adversarial examples, reduced the DL ECG rhythm classification accuracy from 88% to 26%.^15^

Model comprehension and clinical reasoning can improve with scale and prompt tuning, suggesting the potential utility of large language models (LLMs), such as Med-PaLM, for advanced reasoning capabilities in clinical practice. Some clinicians argue that these models remain inferior to more traditional methods of incorporating human interaction, which highlights the need for better evaluation frameworks and method development to ensure safe and effective use of LLMs in clinical settings.^16^

#### Limitations of ML

- Clinical validation of ML methods would necessitate close collaboration with clinicians who understand the system and methods well enough to identify false claims/results. For example, a result may be technically accurate but not clinically useful or meaningful.
- Poor inputs result in inaccurate data, which affect the quality of analysis and reliability of ML techniques.^8^ Models need to be developed using diverse populations, and the methods should be validated in an independent dataset with the goal of generating an unbiased, generalizable model.
- ML and DL algorithms may encounter domain shift issues, where the diagnostic or predictive accuracy is compromised due to variations in data being used to train the computer, data collection methods, or data-capturing devices utilized by the organization. Therefore, external validation by expert clinicians and subject-matter experts plays a key role in ensuring reliability of AI systems.^17^ APPs managing patients across the spectrum of disease processes can offer important perspectives when these AI systems are being developed.
- “Black box” models generated by AI may not be readily understandable by clinicians and/or the engineers who create them.^18^
- Predictions generated by “black box” systems may be inaccurate due to flawed training data, which poses significant challenges.^9^ Repeatability and reproducibility of results by independent research teams are key.

### Natural language processing (NLP)

NLP models aim to equip computers with the ability to comprehend and process human language in spoken or written format.^19^ NLP holds significant promise for streamlining data capture from the EHR that does not exist in discrete fields. NLP is also useful in generating patient education and optimizing provider-patient communication. Building upon this process, LLMs have the capability to produce language that is also contextually relevant to a specific population. LLMs can be used for streamlining administrative tasks and can, for example, enhance data collection methods for quality improvement projects or research. LLMs can also be used with population-specific, evidence-based clinical guidelines to help guide clinical decision-making.

#### Generative AI versus NLP

Generative AI models are more dynamic than NLP models and can create original content, such as distinctive and high-caliber images or unique educational materials that are focused on an individual's specific CV diagnosis. This capability is important for the digitization of EHRs, as generative AI models utilize NLP to create and condense patient notes, extract data from unstructured clinical text, and automate EHR data entry, thereby saving significant time for clinicians.^9^,^19^,^20^ Berman and colleagues used the open-source Canary NLP system to create a highly precise model and study its ability to detect five major CV comorbidities—hypertension, dyslipidemia, diabetes, coronary artery disease, and stroke/transient ischemic attack—within a vast EHR database against physicians' ability to extract such data manually.^21^ Utilizing the NLP models, the sentence- and note-level sensitivity, specificity, and positive predictive value for the modules were greater than 85%. Accuracy was highest for conditions for which diagnostic criteria are straightforward, such as diabetes and hypertension, and slightly lower for conditions involving more diagnostic complexity, such as myocardial infarction and embolic stroke.

Many APPs are involved in their organizations' data management for quality improvement and clinical research projects. Idnay and colleagues found that NLP systems are effective by impacting the time efficiency, workload demands, and recruitment process for clinical research.^22^ The systematic review of 11 studies showed that NLP systems can effectively exclude patients who do not meet eligibility criteria, achieving a 100% negative predictive value; although some ineligible patients were misclassified as eligible, eligible patients were not excluded.^22^ Seven of these studies noted that using NLP significantly reduced the time and workload required for eligibility prescreening with potential for additional reduction as the number of protocols and/or patients increase. Overall, the findings suggest that NLP systems are helpful in clinical research eligibility prescreening but require further investigation to determine their real-world applicability and reliability to produce meaningful data.

#### Limitations of NLP

- Thorough responses to specialized diagnosis-specific queries are difficult to generate with some AI models due to their restricted grasp of domain-specific knowledge on which they have not been trained.^19^ For example, LLMs are generally trained on publicly accessible data that include minimal clinical documentation.
- Training LLMs via the input of textbook-based instruction often fails to capture the intricacies of real-life patient scenarios (that is, the human element in the art of medicine).
- NLP-generated suggestions for practice may fall short of clinical decisions generated through a team's interprofessional collaboration.^19^,^21^ Clinician accountability and well-being may be affected.

### Rule-based expert systems (RBSs)

RBSs mimic human decision-making processes by using knowledge bases and predefined rules, generally defined as “if-then” statements, to diagnose diseases and recommend treatment. RBSs are best suited for straightforward tasks related to basic guidelines or standard practices. Chatbots often use RBSs. Chatbots are computer systems that detect keywords and language identifiers to trigger prewritten responses. If chatbots are equipped to use AI technologies such as ML, NLP, and RBSs, they can, for example, analyze symptoms from collected data, understand a clinician's question, and then automate a response. This combination of AI is well established in clinical practice.

RBSs offer transparency and interpretability, allowing clinicians to understand the RBS's decision-making process to validate its recommendations against established evidence-based clinical guidelines. Applying this concept, Brown and colleagues developed a straightforward RBS application that used a structured tool to integrate relevant established CV disease prevention guidelines and expert consensus statements to make related recommendations to clinicians in CV care of cancer survivors.^23^ Seto and colleagues adopted a comparable strategy in a distinct context.^24^ They developed an RBS for a HF mobile phone-based telemonitoring system that generated alerts and instructions based on the patient's weight, BP, heart rate, and symptoms.^24^ Findings of the trial suggested that the implementation of the rule set was linked to a notable improvement in self-care practices and the clinical management of HF.^24^

RBSs have the potential to detect HF among high-risk patients earlier using an AI-Clinical Decision Support System (AI-CDSS).^8^,^25^,^26^ For example, Choi and colleagues developed an AI-CDSS that achieved an accuracy rate of 98% for HF diagnosis, surpassing that of non-HF specialists (76%).^25^ Similarly, Attia and colleagues applied AI-CDSS to ECG data and found that it could serve as an effective screening approach for detecting left ventricular dysfunction, even in asymptomatic individuals.^27^ Boehmer and colleagues conducted the MultiSENSE study to create and validate a device-based diagnostic algorithm, HeartLogic, for forecasting HF events.^28^ Persons with implanted cardiac resynchronization therapy defibrillators were enrolled for chronic ambulatory data collection.^28^ The alert algorithm, which incorporated heart sounds, respiratory rate, tidal volume, heart rate, and physical activity, surpassed both primary endpoints, exhibiting 70% sensitivity and an unexplained alert rate of 1.47 per patient-year.^28^ Overall, the algorithm emerged as a sensitive and timely predictor of impending HF decompensation.^28^

#### Limitations of RBSs

- Unlike ML that can learn from experiences, RBSs do not have a learning component; therefore, novel or complex scenarios would require manual intervention to incorporate new knowledge into the system.
- RBSs lack the adaptability and learning elements of generative AI systems. Their functionality is thus limited by their predefined rules and database. This limitation would be problematic in complex CV care, which must adapt to clinical scenarios that do not consistently follow clear rules and patterns.
- For reliability, in training chatbots, the rules need to be derived from data, books, research studies, and webpages that are vetted by subject-matter experts.
- Hybrid approaches combining RBSs with ML aim to leverage transparency with adaptability for improved decision support.

### Physical robots using AI

Integrating robotic and AI technologies offers numerous advantages in cardiology. Examples of applications include the early detection of CV risk, precise diagnosis of complex congenital structural heart abnormalities, tailored HF treatment, surgical precision during valve replacement, continuous monitoring for abnormal heart rhythms, and the ability to procure samples for predictive and prognostic analysis of CV disease.^29^-^32^ However, ethical considerations exist, such as the absence of human interaction, risk of errors and malfunctions, restricted accessibility, reliance on data accuracy, and regulatory and legal hurdles.^29^

The integration of AI and robotics can improve the speed and quality of care. AI use in robotic surgery is primarily performed by extensive data collection from sensors and images and can be categorized into two main areas: robotic autonomy and surgical assessment/feedback. For example, AI contributes to intelligent assistance and robotic autonomy during cardiac valve repair by enhancing the surgical field, recognizing native tissues, delineating instruments to use, providing tactile feedback, and automating steps progressively. ML aids in robotic surgical assessment and feedback by recognizing workflow patterns and gestures, assessing intraoperative conditions, and measuring surgical complexity; NLP systems can enhance realism in simulation environments when training APPs novice to CV practice.^32^,^33^

Robotic process automation (RPA) is defined as a software robot that mimics human actions, whereas AI is the simulation of human intelligence by machines. RPAs handle tedious, repetitive tasks such as billing, patient and staff scheduling, and patient records management to improve efficiency and accuracy. RPA applications include remote patient monitoring using implanted or wearable devices as well as simplification of insurance eligibility checks and claims processing.^34^,^35^ Automation assists organizations in predicting staffing and supply/equipment needs. RPA can be trained to compose clinical documentation from various sources such as EHRs, clinical assessments, or past correspondence and to utilize speech recognition NLP during consultations to ease the administrative workload for clinicians.^36^,^37^ Additionally, AI techniques have been suggested for extracting data from handwritten medical forms.^38^

Overall, robotic technology can enhance efficiency, accuracy, and consistency in healthcare delivery. The blending of AI and RPA technologies has the potential to enhance job satisfaction among APPs by allowing them to maintain focus on direct care tasks.^39^ While academic hospitals are exploring these innovations, it is necessary to perform trials that evaluate AI's impact on quality of care and utility to clinicians and care teams.^40^,^41^

#### Limitations of robots

- Frontline workers have expressed concerns over robots and other technological advances, fearing potential drawbacks such as inaccurate diagnoses, compromised patient data privacy, and staff reductions under the guise of innovation and efficiency.
- A commonly held belief is that AI technology cannot substitute for the expertise and care provided by human medical professionals. In a survey, Duke University physicians voiced concerns that AI models could worsen existing care issues, particularly relating to bias.^41^ One respondent emphasized the lack of understanding regarding how to assess algorithm performance, especially concerning certain racial and ethnic groups.

## APPLICATION OF AI TO CLINICAL PRACTICE

**FIGURE 1.**
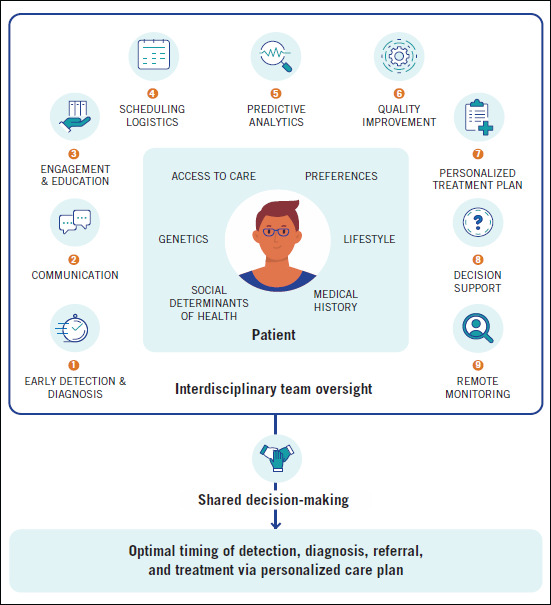
Application of AI in APP clinical practice © Massachusetts General Hospital Institute of Health Professions 2025. The content in this original figure was conceived by Amy Simone with assistance from the other authors; content then underwent graphic design by Megan Wilcox (MGH Institute of Health Professions).

Table 1 provides a summary of the AI models described throughout this article, and Figure 1 illustrates some of the ways in which these AI technologies can be incorporated into clinical practice for the benefit of the APP, the patient, and the overall healthcare system. **Box 2** bookends the article's discussion of AI models and their application to APPs' clinical practice through its presentation of *Case study #2*, an additional exemplar examining use of AI in the care of a patient with CV disease.

**TABLE 1.** Summary of AI terminology^2^

Type of AI	Description	Examples
ML	AI subfield that enables computers to analyze data autonomously, uncover patterns within datasets, apply patterns to new data, and execute computational tasks with greater efficiency than humans	Prediction models (readmission, CV risk, mortality, clinical trial screening eligibility)
DL	ML subset that teaches computers to process data like the human brain by recognizing complex patterns in images, text, sounds, and other data to forecast patterns and make predictions	Algorithm detection devices for atrial fibrillation or sudden cardiac death, prediction of clinical trial research outcomes
Generative AI	Dynamic system capable of analyzing data, making predictions, and generating original content	Distinctive, high-caliber images or educational materials that are unique to patient's diagnosis
NLP	Computers with the ability to comprehend human language in spoken or written format	Digitization of EHRs, comorbidity detection
LLM	Artificial neural networks that learn language patterns, grammar, and context	Translation of educational materials into different languages, compilation of multiple journal articles into a summary
RBSs	Programs that mimic human decision-making processes to diagnose diseases and provide treatment recommendations using predefined rules and knowledge bases	Treatment recommendations, early disease detection
Physical robots	Robots used for surgical precision and continuous monitoring to enhance the ability for precise diagnosis and predictive analysis	Surgical autonomy: automated dispensing cabinets, barcode medication administration, and closed-loop electronic medication management systems
RPA	Enables AI to handle tedious, rule-based repetitive tasks	Remote patient monitoring, insurance reimbursement systems, clinical note transcription

## CHALLENGES IN APPLICATION OF AI ACROSS THE CARE CONTINUUM

The goals of AI use in healthcare are to predict or diagnose disease based on data input, customize treatment strategies, monitor individual outcomes, prognosticate trends across populations or disease-specific cohorts, and enhance provider efficiency, among other aims. Although use of AI is becoming more mainstream in healthcare delivery, APPs should consider several important, real-world issues when interacting with AI and integrating these technologies into clinical and administrative activities along the care continuum. Balagurunathan and colleagues have described several challenges in applying AI to patient care, such as the availability of curated datasets for model building, the ability of clinicians to interpret or comprehend the information produced by AI systems, and reliability and reproducibility of AI methods for routine clinical use.^42^ To expand upon common challenges and concerns involved with the application of AI, we offer a series of questions for APPs to consider.

### Data acquisition

- What resources are used to train the AI computer? Are they human- or computer-generated? How are training resources validated for reliability and accuracy?
- How are data collected? Are the used data collection methods secure? Is the AI developer transparent regarding how the data are collected?
- Does the technology use human- or machine-linked input? What is the potential margin for error/subjectivity?
- What personnel/professions are involved in data collection/development of AI? Is the AI method appropriate for the topic being addressed or use case?
- What is the potential for AI-related job displacements, organizational restructuring, or personnel retraining?

Techniques used by AI to acquire data may lack transparency, posing difficulties in understanding their reliability and accuracy. It may be unclear, for example, which data an AI tool is using, how representative those data are, and if biases are present. EHR data, for instance, may not be applicable to the general population, and their use as a data source in an AI tool could result in unjust treatment or erroneous decisions.^43^-^45^ Transparency can be achieved by explicit model definition followed by model validation.

AI may be limited in its ability to adopt a comprehensive approach to clinical decision-making, potentially overlooking the input of both psychological and social determinants of health. Therefore, it is important that clinicians know how AI models are developed to verify credibility of the outputs. Inaccurate predictions resulting from flawed training data used to develop the AI model pose significant reliability concerns. Overcoming these challenges necessitates collaboration among a variety of clinical and administrative professionals working with specific patient populations. Further, sustainable solutions will require collaboration among technology developers, researchers, policymakers, and clinicians to ensure the design and implementation of responsible, ethical, and safe AI methods and tools for clinical practice. Clinicians should investigate whether the AI technology will apply an algorithm that could compound existing inequities in their patient population.

### Data recognition

- What platforms are being used? ML, DL, NLP, RBSs, and/or RPA?
- In which groups is the use of AI recognition models appropriate or inappropriate? Which cohorts/trends will be used in the data recognition?
- Who has access to data? Patients, providers, healthcare organizations, insurance companies, researchers, patient advocacy organizations, etc.?

A nationwide call has been made for companies to set standards around the development of AI, but no definitive standards have been universally adopted. There is growing concern among healthcare organizations that, in the absence of regulation, some technology companies may prioritize profit over the privacy and safety of patients. Additional concern has been reported in the media over how AI can be weaponized by hackers and other bad actors.^46^ Health systems will need to consider the cost, complexity, and function of new AI technology and its implementation. Recently, comprehensive return on investment calculators have emerged that can guide health systems in assessing the financial and non-financial benefits of AI for patient management.^47^

### Diagnosis

- What algorithms were used to generate probable clinical diagnoses? Are those algorithms comprehensive and representative of the population being assessed?
- What is the repeatability and reproducibility of the AI model's results or data output?
- What cohorts/trends were used in the development of models to generate diagnoses or inform management decisions? Are they current?

AI can significantly enhance the speed and accuracy of CV risk prediction and disease detection. ML techniques currently used by many CV tests and procedures have a greater impact on an APP's ability to practice when DL methods are applied. For example, echocardiograms assess parameters like pressures and volumes that lead to various cardiomyopathies, ECGs detect life-threatening heart rhythms or left ventricular dysfunction, and cardiac implantable devices recognize impending acute HF exacerbation.^27^,^48^,^49^ Weng and colleagues demonstrated the usefulness of ML models in predicting CV disease development, with the most highly performing examined ML model correctly predicting an additional 7.6% of patients who ultimately developed CV disease over 10 years when compared with established risk prediction algorithms.^50^ In medical decision-making, APPs can bring together information gathered from patients, such as preferences and concerns, with clinical data to develop individualized, evidence-based treatment plans.

### Management

- How is information disseminated regarding management?
- Is the AI system secure? Is it linked to an organization or a proprietary, national, or global database?
- What algorithms are used in treatment recommendations? Are they proprietary or based on national or global agency guidelines? Do they incorporate insurance plan coverage considerations?
- What actions does the tool take regarding dissemination and implementation?
- How are outsourcing recommendations generated (for example, recommendations for surgical consult, case management, or palliative care)?
- Will information or guidance be transmitted directly to patients or will input/validation from the provider be required first?

AI algorithms are intended to streamline administrative processes, optimize resource allocations, and perform general administrative operations such as billing, staffing, and composing clinical notes. Development of robust AI in these healthcare processes relies on the availability of precise, high-quality healthcare data. To achieve broad adoption, data will need to be shared widely, requiring deidentification and increased considerations for patient confidentiality and privacy.^51^

### Patient privacy, engagement, and adherence

- How are patient data integrated into AI algorithms and rules to generate patient-specific educational materials?
- Will patient data be used to build future AI algorithms? How will patient identifiers be kept secure?
- Does the AI method used create biases based on demographics or social determinants of health?

AI applications provide new avenues for personalized healthcare management or precision medicine. AI-driven systems have the capacity to produce tailored suggestions across a range of domains such as nutrition, mental health, and fitness; however, the validity and efficacy of these methods still require more thorough study.^52^-^56^ For example, Dergaa and colleagues investigated the efficacy of exercise prescriptions generated by OpenAI's Generative Pre-trained Transformer 4 (GPT-4) model and found that although the model was capable of generating a range of safety-conscious programs, it lacked precision in addressing the patient's personal health conditions and goals.^57^ AI strives to increase patient engagement and adherence by enhancing access to exercise prescriptions, diet prescriptions, and surgical and rehabilitation monitoring, but it is not yet globally recommended for replacing personalized and health condition-specific prescriptions provided by trained professionals.^57^,^58^ AI-based regimens tend to be conservative and lack specificity in recommendations that can impact a patient's motivation and adherence. Without real-time feedback and responsive program adjustments, which an APP can provide through virtual or in-person follow-up visits, these challenges are further compounded.^57^

### Surveillance

- How will trending be done regarding specific cohort-related Diagnosis-Related Group (DRG)/International Classification of Diseases (ICD)?
- How will trending be used to monitor at the individual, provider, and practice group levels?
- How will trending be used by the Centers for Medicare and Medicaid Services (CMS) and other insurance providers?

Ethical, social, and technical concerns often arise when applying AI techniques to clinical practice. Patient data security concerns often arise when AI uses EHR data. Ethically, AI systems need to preserve patient confidentiality while providing data to build artificial neural networks.^59^ There are perceived reliability and trust issues in AI-driven decision-making. Therefore, APPs must learn to use AI responsibly across all clinical settings to protect patient privacy while monitoring individual patient outcomes among population-specific pooled data.

### Evaluation

- What quality control measures are in place? Who will evaluate and perform quality control (for example, the AI proprietor or a national/governmental agency)?
- Does the AI method used need adjustments to reflect current APP practices?
- How will new AI methods be developed based on quality control recommendations?

The evaluation process is important for any new AI system to determine if the methods or algorithms work well for the intended purpose and if they are based on the most current evidence or studies available. For example, the inclusion of APPs on insurance group panels was delayed due to CMS being slow to recognize APPs as independent and/or as contributing providers of healthcare management. AI systems must continuously learn or be updated to reflect current national standards. Balagurunathan and colleagues offer a checklist of criteria, adapted below, that can be used to improve the reliability and transparency of AI methods:

✓ Diverse cohort of patient records for model training, achieved either through use of centralized or federated/distributed learning models that use silos of different data sources
✓ Use of independent data cohort for testing, preferably in a distributed setting with diverse patient types
✓ Transparency of deep network model architecture with confidence levels in its decisions
✓ Ethically appropriate use of AI methods with some level of oversight
✓ Assessment of reproducibility of AI models with test-retest type studies
✓ Model transparency that discloses the architecture, datasets, and trained weights for the network
✓ Quality assurance program for implementation and continuous performance monitoring.^42^

## CONCLUSION

AI systems can be used by APPs to augment their knowledge base for optimal clinical decision-making and to enhance communication between patients and the entire healthcare team. AI methods hold enormous promise in CV care and are already demonstrating value in driving efficiency, supporting complex analyses, generating predictive models, and empowering clinicians with more tools to screen, diagnose, and manage patients with increasingly personalized approaches.

AI methods are here to stay, so it is imperative for APPs to gain an understanding of AI technologies used in healthcare, recognize their potential benefits and limitations, and support the development and evaluation of reliable and reproducible models that add meaningful clinical value and efficiency to the care continuum. Fortunately, frameworks and protocols exist to help integrate equity, diversity, and inclusion principles and practices in the design, development, and implementation of AI technologies in healthcare.^60^ APPs have the opportunity to embrace and utilize AI in their clinical practice to optimize quality and safety of care. This article has provided examples and resources of how to incorporate AI into clinical practice. We recommend accessing “Navigating the Responsible and Ethical Incorporation of Artificial Intelligence into Clinical Practice,” a resource from the Federation of State Medical Boards, for further reading.^61^

## BOX 1. Case study #1

This case study illustrates the aspects of AI models that can be incorporated into clinical practice for patients to navigate between facilities and providers. Below the case study, a list of ways in which AI can augment patient care is provided; the example (EX) codes cited throughout the case text refer to this list.

**Case**

Mrs. Smith is a 78-year-old Black woman who recently presented to an ED with chest pain and shortness of breath. She underwent an echocardiogram, which revealed significant aortic stenosis, and received supportive care. Further testing ruled out myocardial infarction or significant coronary artery disease. She was discharged home with instructions to follow up with her primary care provider (PCP).

Mrs. Smith resides in a rural community with limited access to advanced heart and vascular services. Her PCP referred her to a comprehensive valve center approximately 100 miles away that offers an echo surveillance application (EX1, EX2) as a quality initiative to ensure patients with significant valvular heart disease are referred for evaluation. The application assessed her aortic valve disease and, utilizing predictive analytics (EX3), indicated that she was likely to progress rapidly to critical aortic stenosis.

Mrs. Smith's PCP was notified (EX4) that she met criteria for significant aortic stenosis based on the latest American College of Cardiology (ACC)/American Heart Association (AHA) guidelines. Mrs. Smith received a phone call from an APP at the valve center explaining the circumstances and that her PCP had approved a referral (EX4) for further evaluation. Education materials were generated that were appropriate to Mrs. Smith's health literacy and education levels (EX5) and sent to her prior to the appointment. Mrs. Smith underwent a CT scan, which applied a predictive modeling application (EX3) and identified significant risks (EX6, EX7) for transcatheter aortic valve replacement. The patient was engaged in a shared decision-making conversation by the care team using tools personalized for Mrs. Smith's unique anatomical considerations and health literacy levels (EX8). Mrs. Smith ultimately underwent surgical aortic valve replacement with an excellent result.

**Ways AI can augment care**

- EX1: Mitigate disparities
- EX2: Improve access to care
- EX3: Apply predictive analytics to diagnostic tests
- EX4: Ensure swift referral and operational efficiencies
- EX5: Provide personalized education resources
- EX6: Strive to mitigate complications
- EX7: Tailor treatment plans for optimal clinical quality and outcomes
- EX8: Generate an individualized shared decision-making toolkit

## BOX 2. Case study #2

This case study illustrates the aspects of AI models that can be incorporated into clinical practice. Below the case study, a list of ways in which AI can augment patient care is provided; the example (EX) codes cited throughout the case text refer to this list.

**Case**

Mr. Garcia is a 52-year-old Latino man referred to an advanced HF clinic for newly diagnosed nonischemic cardiomyopathy following a recent hospitalization for decompensated systolic congestive HF. AI flagged Mr. Garcia's chart for APP review (EX1) prior to his visit because the medical regimen Mr. Garcia was prescribed was identified as suboptimal. Prior to his appointment, a buccal swab collection kit and educational materials in his preferred language (Spanish) were sent to him so that a pharmacogenetic test could be performed (EX1, EX2, EX3, EX4). During the appointment, AI was implemented to translate Spanish to English in real time (EX4) and help construct the clinic note (EX5), allowing the APP to focus on building rapport and ensuring an ideal patient experience despite the language barrier. AI provided a personalized medical regimen of guideline-driven medical therapy (EX1), in view of Mr. Garcia's genetic profile and predicted levels of response. At the visit, his echocardiogram, ECG, and biomarkers were assessed by AI (EX2), which determined him to be at elevated risk for sudden cardiac death via ventricular arrythmia; therefore, an internal cardiac defibrillator (ICD) was recommended. A shared decision-making (EX6) toolkit was utilized by the HF APP, and Mr. Garcia agreed to ICD implantation and placement of an invasive pulmonary artery pressure device. Mr. Garcia did well clinically and underwent ongoing remote monitoring. The care team was alerted several months later that he was becoming fluid overloaded (EX7, EX8) and would benefit from outpatient IV diuretics to avoid hospitalization and overt decompensation. After 12 months, an AI application automatically reviewed Mr. Garcia's clinical progress and alerted the APP (EX1) that Mr. Garcia would be a reasonable candidate for a left ventricular assist device as a bridge to heart transplantation.

**Ways AI can augment care**

- EX1: Tailor treatment plans for optimal clinical quality and outcomes
- EX2: Apply predictive analytics to diagnostic tests
- EX3: Strive to mitigate complications
- EX4: Provide personalized education resources
- EX5: Utilize generative AI in clinic note construction
- EX6: Generate an individualized shared decision-making toolkit
- EX7: Decrease risk for inpatient hospitalization
- EX8: Prevent complications
